# Spermidine-induced recovery of human dermal structure and barrier function by skin microbiome

**DOI:** 10.1038/s42003-020-01619-4

**Published:** 2021-02-19

**Authors:** Gihyeon Kim, Misun Kim, Minji Kim, Changho Park, Youngmin Yoon, Doo-Hyeon Lim, Hyeonju Yeo, Seunghyun Kang, Yeong-Geun Lee, Nam-In Beak, Jongsung Lee, Sujeong Kim, Jee Young Kwon, Won Woo Choi, Charles Lee, Kyoung Wan Yoon, Hansoo Park, Dong-Geol Lee

**Affiliations:** 1 Department of Biomedical Science and Engineering, Gwangju Institute of Science and Technology (GIST), Gwangju, Republic of Korea; 2 R&I Center, COSMAX BTI, Pangyo-ro 255, Bundang-gu, 13486 Seoungnam-si, Gyeonggi-do Republic of Korea; 3 Genome and Company, Pangyo-ro 253, Bundang-gu, 13486 Seoungnam-si, Gyeonggi-do Republic of Korea; 4 Graduate School of Biotechnology and Department of Oriental Medicine Biotechnology, Kyung Hee University, 17104 Yongin, Republic of Korea; 5 Dermatology Laboratory, Department of Integrative Biotechnology & Biocosmetics Research Center, College of Biotechnology and Bioengineering, Sungkyunkwan University, 16419 Suwon City, Gyeonggi-do Republic of Korea; 6 The Jackson Laboratory for Genomic Medicine, Farmington, CT 06032 USA; 7 Wells Dermatology Clinic, 583 Shinsa-dong, Gangnam-ku, Seoul, Republic of Korea; 8 Department of Life Science, Ewha Womans University, 03760 Seoul, Republic of Korea; 9 The First Affiliated Hospital of Xi’an Jiaotong University, 710061 Xi’an, China; 10 Department of Biotechnology, Hoseo University, Asan, 31499 Republic of Korea

**Keywords:** Microbiology, Cell biology, Medical research

## Abstract

An unbalanced microbial ecosystem on the human skin is closely related to skin diseases and has been associated with inflammation and immune responses. However, little is known about the role of the skin microbiome on skin aging. Here, we report that the *Streptococcus* species improved the skin structure and barrier function, thereby contributing to anti-aging. Metagenomic analyses showed the abundance of *Streptococcus* in younger individuals or those having more elastic skin. Particularly, we isolated *Streptococcus pneumoniae*, *Streptococcus infantis*, and *Streptococcus thermophilus* from face of young individuals. Treatment with secretions of *S. pneumoniae* and *S. infantis* induced the expression of genes associated with the formation of skin structure and the skin barrier function in human skin cells. The application of culture supernatant including Streptococcal secretions on human skin showed marked improvements on skin phenotypes such as elasticity, hydration, and desquamation. Gene Ontology analysis revealed overlaps in spermidine biosynthetic and glycogen biosynthetic processes. *Streptococcus*-secreted spermidine contributed to the recovery of skin structure and barrier function through the upregulation of collagen and lipid synthesis in aged cells. Overall, our data suggest the role of skin microbiome into anti-aging and clinical applications.

## Abstract (web summary)

Kim et al. show that several *Streptococcus* species improve the structure and barrier function of human skin. They find that *Streptococcus*-secreted spermidine accelerates the recovery of skin structure and barrier function by increasing collagen and lipid synthesis in aged cells. This study suggests the role of skin microbiome for anti-aging.

## Introduction

The human skin has a multi-layered structure composed of various cell types, fibers, lipids, and other components. It is the primary organ to protect the body from the external environment^1^. The skin surface maintains acidic, desiccated, and aerobic environments, whereas sebaceous follicles maintain an anaerobic and lipid-rich environment. Furthermore, the human skin is populated by various microorganisms including fungi, viruses, archaea, and bacteria, which feed on sebum, lipids, and keratin^2^. The most abundant microorganisms are bacteria, which produce several metabolites and affect the proliferation and immune responses of the skin^3–5^. Numerous non-pathogenic bacteria living on the skin have been studied through culture isolation methods, and their characteristics have been identified by molecular analysis. Recently, high-throughput deep sequencing of the bacterial 16S ribosomal RNA (rRNA) has enabled researchers to analyze the skin microbiome (skin microbiome means bacteria living on the skin or genomic information of them)^3,6^ and to investigate the link between skin health and the skin microbiome. Many studies revealed that the marked changes in the skin microbiome can alter skin conditions and cause diseases such as acne, psoriasis, and atopic dermatitis^7–9^.

Skin aging is caused by a combination of intrinsic factors, such as changes in hormones, cellular metabolism, and immune responses, and external factors, such as exposure to pollutants and ultraviolet rays^9–12^. They induce major physiological and physical changes in the skin, such as sagging, wrinkles, dryness, and low elasticity^10^. Changes in the lipid composition, sebaceous secretion, pH, and hydration of the skin during aging can alter the skin microbiome composition which is colonized at birth^13–19^.

The effects of the gut microbiome on skin health were also reported. The absorption of probiotics through the intestinal tract has been shown to improve skin health, known as gut–skin axis^20,21^. However, these studies have only looked at the indirect role of the microbiome in improving skin conditions; direct evidence on the impact of the skin microbiome on skin health should be evaluated.

Here, we sought to investigate the direct link between skin health and skin microbiome in terms of aging. We designed a stepwise study to identify the components of the skin microbiome and their roles in controlling skin conditions. We observed that *Streptococcus* colonies are enriched in the facial skin of young Korean women (20–29 years old) and demonstrated their roles in the improvement of skin health through metagenomic analysis, in vitro assays using human skin cells, clinical analysis, and genomic analysis with validations (metabolic and cellular analyses). Our findings suggest the potential of the *Streptococcus* species to rejuvenate aged skin, thereby providing insights on the possible applications of the skin microbiome in clinical practice.

## Results

### Metagenomic profiling reveals specific microbes related to facial skin aging

To examine the relationship between age and facial skin microbiome, we enrolled 52 healthy Korean women participants (Supplementary Table 1). They were divided into two groups: old (40–53 years old) and young (20–29 years old). The age limit was set to 54 years old to minimize age-related changes in sebum, moisture, and cell proliferation, which could lead to microbial bias due to marked alterations in skin microbiome^14^.

**Fig. 1:**
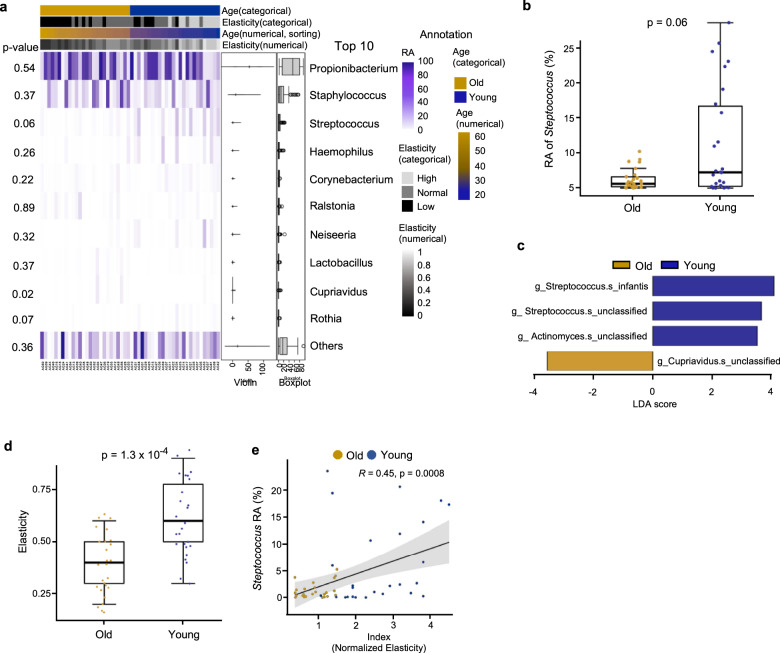
*Streptococcus* species are the potential to anti-aging of skin. **a** Relative abundance (RA) of facial skin microbiome at the genus level including some clinical information. The top 10 most abundant genera are presented. Violin plot and boxplot show the RA of each skin microbiome with the columns sorted by age. **b** Boxplot illustrating the RA of the *Streptococcus* genus in old (*n* = 26) and young (*n* = 26) participants. **c** Taxonomic plot of LDA scores from LEfSe, illustrating the sequenced microbiome that differed significantly at least at the genus level between the old and young group (|LDA score| > 3.5); g, genus; s, species. **d** Boxplot illustrating the elasticity index of the old and young groups. Wilcoxon–Mann–Whitney test and Pearson correlation test were used for statistical analysis. **e** RA of *Streptococcus* versus normalized elasticity index of the old and young groups.

We first identified and quantified the overall facial microbial composition in relation to age as described previously^16,17^ through 16S rRNA sequencing. At the genus level, only *Streptococcus* were more abundant in the young group compared with the old (Fig. 1a, b), and no significant differences were observed for other genera (Supplementary Fig. 2b). In particular, *Streptococcus infantis* (*S. infantis*) was significantly enriched, as well as some unclassified species of *Streptococcus* but to a lesser extent (Fig. 1c). The five most abundant phyla were present in both the young and old groups (Supplementary Fig. 1). The lack of age-related stratification was confirmed by microbial compositional distance measurements (Supplementary Fig. 2a). Based on the metagenomic analysis, we hypothesized that *S. infantis* or some other unclassified *Streptococcus* species could be closely related to facial skin aging.

### Abundance of *Streptococcus* is associated with facial biophysical properties

To confirm *Streptococcus* as a potential major microbe that determines skin aging, we evaluated the biophysical properties of the skin. Because elasticity is an important indicator of facial skin condition and is associated with aging^22^, we investigated the abundance of *Streptococcus* in participants with varying facial elasticity. After measuring elasticity from 0 to 1, we split the elasticity index into low (0–0.3), normal (0.4–0.6), and high (0.7–1). Most of the participants with high elasticity belonged to the young group (Fig. 1d). After plotting the relative abundances of *Streptococcus* against the elasticity-to-age ratio, we obtained a positive correlation as seen in Fig. 1e. The microbial compositional distances did not differ significantly in participants with low or high elasticity (Supplementary Fig. 3a). However, consistent with the age-metagenomic profiling results, *Streptococcus* and *S. infantis* were significantly more abundant in the high-elasticity group than in the low-elasticity group (Supplementary Fig. 3b). A comparison of other variables, such as skin appearance and skin moisture, revealed no dependence with microbial distances (Supplementary Fig. 4a, c, respectively). Specifically, *Streptococcus* and *S. infantis* were abundant in participants with clear skin surfaces based on appearance (Supplementary Fig. 4b). However, skin moisture did not seem to have a part (Supplementary Fig. 4d). These results indicate the potential of *Streptococcus* to improve facial skin elasticity.

### Streptococcal secretions upregulated genes related to skin structure and barrier

A previous study revealed that a pyrimidine compound from a bacterial strain, EPI-7T, had an anti-aging effect on human dermal fibroblasts (HDFs)^23^. Therefore, we hypothesized that the abundant *Streptococcus* facial colonies could secrete compounds related to skin aging. First, we isolated *Streptococcus pneumoniae* (*S. pneumoniae*), *Streptococcus infantis* (S. infantis 1), the other of *Streptococcus infantis* (S. infantis 2), and *Streptococcus thermophilus* (*S. thermophilus*) from the facial skin of young female participants. To demonstrate the functional roles of the secretions from *Streptococcus*, we treated primary HDFs and human epidermal keratinocytes (HEKs) with the supernatant derived from the *Streptococcus* culture (St solution) from the said four strains^24,25^. Prior to the analysis, we ensured that all four St solutions were not toxic to the two cell types (Supplementary Fig. 5a), whereas St solutions could encourage the proliferation of skin cells (Supplementary Fig. 5b). We then treated HDFs and HEKs with up to 10% of the St solution in growth medium to determine the optimal concentration for subsequent experiments (Supplementary Fig. 5d), and 10% was chosen as the optimal dose.

**Fig. 2:**
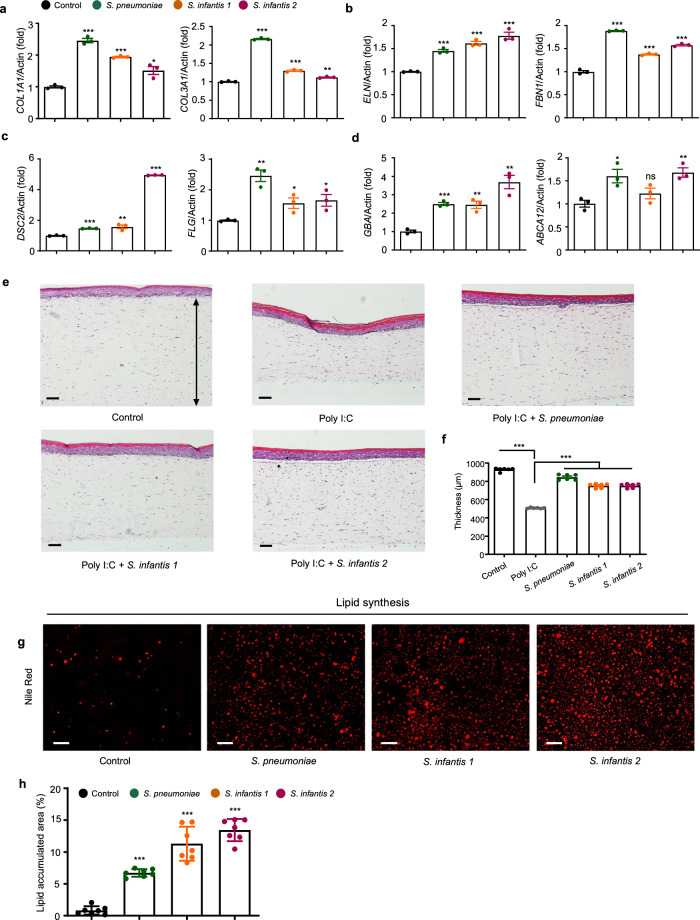
*Streptococcus* growth media improve skin cells with various phenotypes. **a**–**d** Relative mRNA expression levels of **a**, collagen-associated genes important for elasticity in HDFs. **b** Elastic-fiber–associated genes in HDFs, **c** tight-junction–associated genes important for skin barrier function and moisture in HEKs, and **d** lipid barrier-associated genes in HEKs. HDFs human dermal fibroblast, HEKs human epithelial keratinocytes. Expression values are relative to control cells and represent the mean ± S.E. Three technical replicates were done. *COL1A1* collagen type l alpha 1 chain, *COL3A1* collagen type lll alpha 1 chain, *ELN* elastin, *FBN1* fibrillin 1, *DSC2* desmocollin 2, *FLG* filaggrin, *GBA* glucosylceramidase beta, *ABCA12* ATP-binding cassette subfamily A member 12. **e** Micrographs of a skin cell layer in the control and after treatment with Poly I:C, Poly I:C and supernatant of *S. pneumoniae*, Poly I:C and supernatant of *S. infantis 1*, and Poly I:C and supernatant of *S. infantis 2*. 1 μg/mL of Poly I:C was used in each treatment. Arrow line indicates the thickness of the epidermal layer. **f** Corresponding plot of the thickness of skin cell layer, *n* = 6. **g** Nile red staining of HEKs treated with different *Streptococcus*-cultured media. **h** Plot of area of lipid accumulations, *n* = 7. The Student’s two-tailed *t*-test was used to calculate statistical significance. *p < 0.05, **p < 0.01, ***p < 0.001. ns non-significant. Scale bar corresponds to 100 μm. Control is a non-treated condition.

To assess the effects of the St solutions on skin aging, we analyzed the expression levels of genes involved in the dermis structure. The solutions from *S. pneumoniae* and *S. infantis* significantly increased the expressions of collagen type I alpha 1 chain (COL1A1) and collagen type III alpha 1 chain (COL3A1) (Fig. 2a), which are two major components of the extracellular matrix. The major dermal elastic-fiber genes, elastin (ELN) and fibrillin 1 (FBN1) were also considerably induced by the St solutions in HDFs (Fig. 2b). *S. pneumoniae* culture medium exhibited the most potent effect on skin structural components. Multi-dimensional model of the skin layer also showed a thicker epidermal layer after St solution treatment in Polyinosinic:polycytidylic acid (Poly I:C)-induced damaged skin model^26^ (Fig. 2e, f). Additionally, treatment with the St solutions increased the expression levels of desmocollin 2 (DSC2) and filaggrin (FLG), which are responsible for skin barrier function (Fig. 2c). A similar trend was observed for glucosylceramidase beta (GBA) and ATP-binding cassette subfamily A member 12 (ABCA12), which enable the syntheses of lamellar body and ceramide to form the lipid barrier (Fig. 2d). Next, we evaluated the effect of the St solutions on skin lipid synthesis and the consequent maintenance of a healthy barrier in HEKs. The solutions from *S. pneumoniae*, *S. infantis 1*, and *S. infantis 2* increased lipid accumulation, and this was confirmed by the gene expression profiles for lipid synthesis, which involved DSC2, FLG, GBA, and ABCA12 (Fig. 2g, h). The beneficial outcomes were not obtained following treatment with S. thermophilus and other skin microbiome culture medium (Supplementary Fig. 5c, e).

### Streptococcal secretions improved the physiology of human skin

**Fig. 3:**
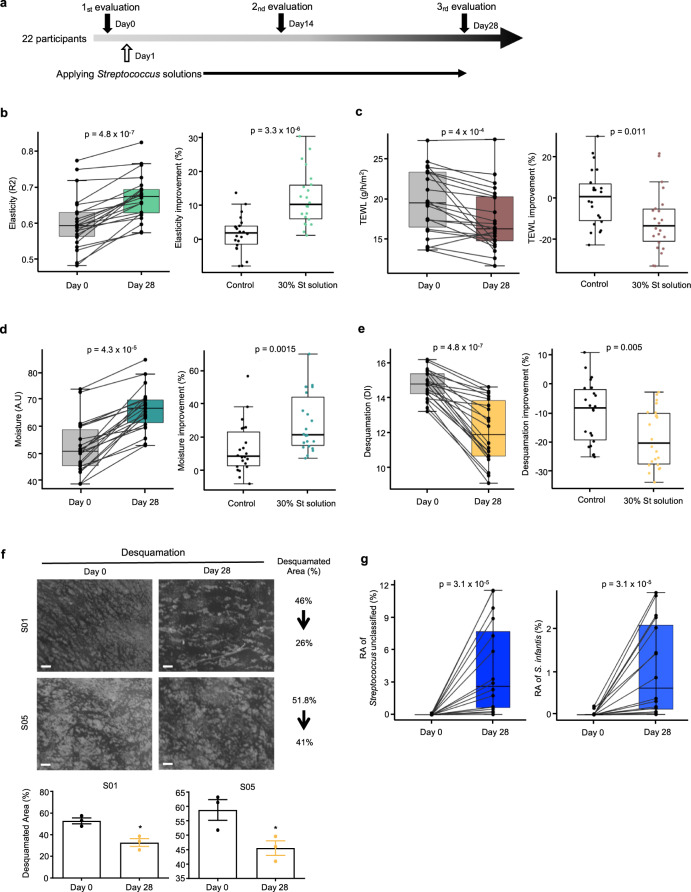
St solutions clinically improve skin phenotypes. **a** Scheme of clinical evaluations. **b**–**f** Boxplots illustrating facial skin parameters on day 0 and day 28 after the application of the St emulsion (left plots), and in control versus St solution-treated individuals (right plots); *n* = 22 per group. **b** Elasticity, **c** TEWL, **d** moisture, **e** desquamation. **f** Direct observation (top) and comparison (bottom) of desquamation on the cheek surface in participants S01 and S05 on day 0 and day 28 of St solution treatment at ×20 magnification. Three technical observations were done. The white area is corneocyte. Scale bar corresponds to 500 μm. **g** Increase in relative abundances of unclassified *Streptococcus* and *S. infantis* on day 28 after application of St solution treatment. The statistical calculation for paired comparison was done using the Wilcoxon signed-rank test, whereas inter-comparison was done using the Wilcoxon–Mann–Whitney test. A.U arbitrary unit, DSC diffuse scattering correction, DI desquamation index.

To confirm whether Streptococcal secretions are clinically effective on human skin, we applied the St solutions on both cheeks of healthy female participants who do not have any skin disease (Fig. 3a and Supplementary Table 2). Phenotype changes were measured on cheeks applied with the control solution and 30% St solution (10% *S. pneumoniae*, 10% *S. infantis 1*, and 10% *S. infantis 2*) at day 0 (day of application) and 28 days after application. After 28 days, significant improvements in various skin phenotypes were observed in cheeks treated with the St solution. Consistent with the increased expressions in the collagen and elastin genes of our in vitro assay, skin elasticity increased significantly from 0.60 ± 0.07 to 0.67 ± 0.07 (mean ± standard deviation; difference 0.06 ± 0.04 or 12.1%; Fig. 3b left), and the extent of which was significantly higher than those in control (Fig. 3b, right), which changed from 0.601 ± 0.07 to 0.61 ± 0.07 (difference 0.01 ± 0.03; Supplementary Fig. 6a). Skin elasticity was maintained at baseline values during the test period in the control-treated cheek. The effectiveness of the St solution on skin moisturization was confirmed by measuring trans-epidermal water loss (TEWL) and horny layer moisture content. TEWL indicates the moisturizing ability and skin barrier function^14,27^. In the St solution-treated cheeks, the TEWL score declined from 19.90 ± 4.23 to 17.44 ± 3.88 g/h/m^2^ (difference −2.45 ± 3.30 or 11.3% reduction) after 28 days (Fig. 3c, left), whereas that in the control did not decrease significantly (difference –0.17 ± 2.82 or 0.21% reduction; Fig. 3c right and Supplementary Fig. 6b). Unsurprisingly, skin hydration in the St solution-treated group was significantly higher after 28 days (66.39 ± 7.73 A.U) compared with the baseline (52.40 ± 0.38 A.U; Fig. 3d left). Even though skin hydration was improved also with control solution, augmenting from 51.83 ± 8.75 A.U to 57.97 ± 7.68 A.U (Supplementary Fig. 6c), the improvement was lower than in the St solution-treated cheeks (Fig. 3d, right).

These improvements in the moisture and lipid composition of the St solution-treated cheeks also led to lower desquamation, which normally increases in older individuals^28,29^. In the St solution-treated cheeks, the desquamation index (DI) was markedly lower after 28 days (12.08 ± 1.794) compared with the baseline (14.77 ± 0.82), corresponding to an 18.4% reduction (difference 2.69 ± 1.46) (Fig. 3e, left). This was confirmed by a reduction in the corneocyte area (Fig. 3f). Meanwhile, the DI of the control solution-treated cheeks decreased by only 9.7% (Supplementary Fig. 6d), which was lower than that in St solution-treated area (difference −1.45 ± 1.56; Fig. 3e, right) despite its significance.

After 28 days, brightness increased in the St solution-treated cheeks, from 72.95 ± 3.24 to 74.24 ± 3.08 (difference 1.784; Supplementary Fig. 6e, center), was greater than that in the control, from 73.24 ± 3.43 to 73.88 ± 3.06 (difference 0.909; Supplementary Fig. 6e, left and right). Skin transparency values were similarly better following the St solution treatment, as indicated by a 6.280% decrease (Supplementary Fig. 6f). We also observed increased abundances in the *Streptococcus* genus (unclassified) and *S. infantis* on the face after 28 days (Fig. 3g). These results altogether suggest that the St solution efficiently improved the structure- and barrier function-related skin physiological parameters. We conclusively suggest that age-related skin microbiome clinically improved the conditions of the multi-skin layer.

### Genomic characteristics and biological pathways are triggered by *Streptococcus*

**Fig. 4:**
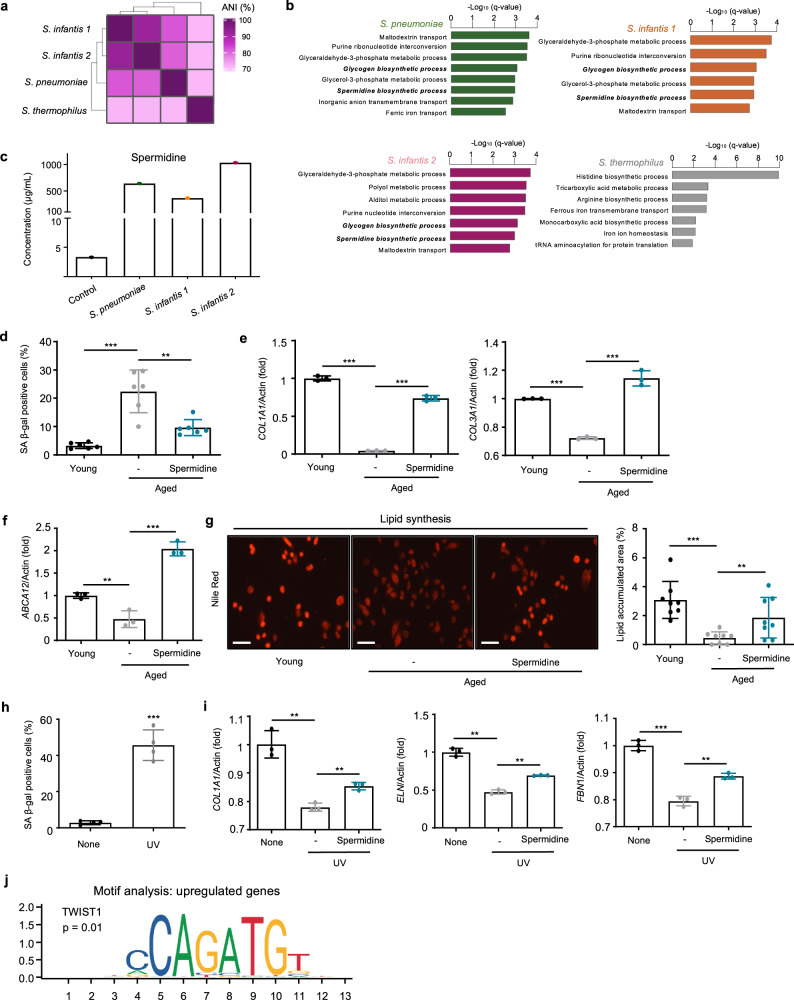
Spermidine contributes to stronger facial skin structure and lipid barrier formation. **a** Average nucleotide identity (ANI, %) among four *Streptococcus* candidates isolated from the face of young participants. **b** Gene Ontology (GO) terms for biological pathways of the genes from candidates with similar ANI scores. Enrichment network pathways were generated using Cytoscape software with ClueGO plug-in, and the overview terms are represented. The calculation for selecting the pathways used a two-sided hypergeometric test, and the false discovery rate was corrected using the Benjamini–Hochberg method. **c** Spermidine concentration in control medium and St solutions. **d** SA β-gal levels in normal, aged, and spermidine-treated aged cells. **e** Relative mRNA expression levels of *COL1A1* and *COL3A1* in normal, aged, and spermidine-treated aged cells. **f** Relative mRNA expression levels of *ABCA12*. **g** Nile red lipid staining of HEKs treated with spermidine. Scale bar corresponds to 50 μm. **h** SA β-gal levels in normal, UV-aged HDFs, and spermidine-treated aged cells. SA β-gal, senescence-associated β-galactosidase. **i** Relative mRNA expression levels of *COL1A1*, *ELN*, and *FBN1* in normal, UV-aged HDFs, and spermidine-treated aged cells. Three technical replicates were done. *COL1A1*, collagen type l alpha 1 chain; *COL3A1*, collagen type lll alpha 1 chain; *ELN*, elastin; *FBN1*, fibrillin 1; *ABCA12*, ATP-binding cassette subfamily A member 12. **j** Motif analysis of the regulatory element in spermidine-treated aged cells. Expression values are relative to young or UV cells and represent the mean ± S.E. Student’s two-tailed *t*-test was used to calculate statistical significance.

To provide a better insight into the genomic and functional characteristics of *Streptococcus*, we analyzed their entire genomes. Consistent with the results of molecular and cellular level assays, *S. infantis 1* and *S. infantis 2* displayed the closest genomic distance, followed by *S. pneumoniae*, whereas *S. thermophilus* showed a low genomic similarity (Fig. 4a). We thus split them into two groups: *S. pneumoniae*, *S. infantis 1*, and *S. infantis 2* in one; *S. thermophilus* in the other. We annotated the genomic fragments using whole-genome analysis, compared their cluster of orthologous groups (COGs) for selecting common genes (Supplementary Fig. 7a, b), and identified their functional roles (Fig. 4b). *S. pneumoniae* was enriched for the following gene ontology (GO) terms: maltodextrin transport, purine ribonucleotide interconversion, and glyceraldehyde-3-phosphate metabolic process; *S. infantis 1*, glyceraldehyde-3-phosphate metabolic process, purine ribonucleotide interconversion, and glycogen biosynthetic process; *S. infantis 2*, glyceraldehyde-3-phosphate metabolic process, polyol metabolic process, and alditol metabolic process; and *S*. *thermophilus*, histidine biosynthetic process, tricarboxylic acid metabolic process, and arginine biosynthetic process. To investigate the biological processes of *Streptococcus* candidates to human skin, we sought all common biosynthetic processes in *S. pneumoniae, S. infantis 1*, and *S. infantis 2*. The overlapping GO terms were the spermidine biosynthetic process and glycogen biosynthetic process.

### Spermidine recovered reduced gene expressions in aged skin cells

We selected spermidine as a potential molecule contributing to skin improvements according to previous studies^30–32^. Spermidine biosynthetic process was also enriched in *S. pneumoniae*, *S. infantis 1*, and *S. infantis 2*. Prior to cell-based assays, we confirmed the existence of spermidine in the St solutions using mass picking and calibration curve. As expected, the concentration of spermidine was higher in St solutions from *S. pneumoniae* (635.3 μg/mL), *S. infantis 1* (361.3 μg/mL), and *S. infantis 2* (1026.9 μg/mL) compared to the media (3.3 μg/mL) (Fig. 4c and Supplementary Fig. 8). To investigate the role of spermidine in skin aging, we used aged cells (see Methods for details) and observed the molecular changes induced by spermidine. We confirmed that treatment with spermidine reduced the senescence-associated β-galactosidase (SA β-gal) in aged HDFs (Fig. 4d and Supplementary Fig. 9)^33^. Spermidine also increased the *COL1A1* and *COL3A1* levels compared with non-treated aged cells (Fig. 4e). Interestingly, the mRNA level of *COL3A1* was higher than those of young cells. We found that spermidine induced lipid synthesis with increased mRNA levels of *ABCA12* (Fig. 4f, g). We also observed similar outputs from the recovery of aging induced by UV which is a major external factor of skin aging^34^. UV-aged HDFs secreted greater levels of SA β-gal than the control (Fig. 4h). Further, UV-aged skin cells expressed lower levels of *COL1A1*, *ELN*, and *FBN1* genes compared with control cells. Interestingly, spermidine recovered the reduced expressions of these genes (Fig. 4i). Furthermore, upon the examination of the regulatory sequences in promoters of genes upregulated upon spermidine treatment, one of the sequence motifs corresponded to the TWIST1 motif, suggesting that spermidine may act as a transcriptional activator (Fig. 4j). Additionally, we discovered that *S. pneumoniae* and *S. infantis*, the effective skin microbiome in this study, exhibited greater growth in presence of spermidine compared with the non-spermidine conditions, whereas *S. thermophilus* showed the opposite growth trend (Supplementary Fig. 10).

## Discussion

The facial skin microbiome formed at birth changes gradually with aging, and any imbalance in their composition is closely related to skin diseases, such as atopic dermatitis, psoriasis, and acne^7,16,19,35,36^. *Staphylococcus aureus*, *Cutibacterium acnes*, and *Streptococcus pyogenes* (group A *Streptococcus*) are examples of pathogenic bacteria related to acne and other infectious skin diseases^37–39^. Depending on age, the composition of the skin microbiome can be changed^13–15^. However, few papers investigated the role of the skin microbiome in the aging of skin^35^, so the effect of the specific microbiome on skin phenotype has remained largely unknown. Therefore, we conducted a stepwise analysis to determine the facial skin microbiome related to aging and to investigate their roles in skin aging.

Metagenomic analysis revealed specific differences in the facial skin microbiome of old and young participants. There were no differences of abundance among the top five most abundant phyla namely, Actinobacteria, Firmicutes, Proteobacteria, Cyanobacteria, and Bacteroidetes. Our result contrasted with a previous report on fewer Actinobacteria and more Bacteroidetes in individuals older than 60^17^. This discrepancy could be explained by the different age range of older participants enrolled in our study. Similarly, there was no significant difference in abundance in the skin microbiome among the top 10 genera. *Streptococcus* was more abundant in young participants, particularly *S. infantis*. Indeed, four *Streptococcus* candidates, i.e., *S*. *pneumoniae*, *S. infantis 1*, *S. infantis 2*, and *S. thermophilus* were isolated through culture from young participants. *S. pneumoniae* is pathogenic in the respiratory tract but not on the skin^40^. Thus, isolated *S*. *pneumoniae*, *S. infantis 1*, *S. infantis 2*, and *S. thermophilus* are possible candidates contributing to facial skin aging.

Next, we investigated the role of the *Streptococcus* candidates in improving skin phenotypes using in vitro cell assays. The secretions of certain *Streptococcus* species were associated with more elastic skin structure, stronger skin barrier, and more moisturized skin through increased mRNA expressions^34,41–47^. We believe that the increased mRNA expressions of various genes related to skin well-being observed in in vitro screening could explain the mechanism underlying these skin improvements^48^. Additionally, the recovery of the damaged skin layer and increased lipid synthesis of skin cells supported the effects of Streptococcal secretions to improve skin structure and the skin barrier function^49–51^. Then, we demonstrated those improvements in human skin layers showing consequent outcomes.

The importance of microbial secretions emphasized at the start-line of our study needed a genomic approach to identify possible microbial secretions^52–54^. The genomic distances of *S. pneumoniae, S. infantis 1*, and *S. infantis 2* suggested that the effective *Streptococcus* candidates shared similar genomic characteristics, which was further confirmed by having common biosynthetic pathways, such as those for spermidine and glycogen. Based on previous studies, we extrapolated that spermidine could be related to skin rejuvenation^30–32^. Spermidine is a key precursor in adipogenesis and lipid synthesis^30^, as well as associated with cell viability and autophagy^55,56^. Our analysis showed spermidine can increase the expression of collagen and elastin, as well as the synthesis of lipids in aged skins based on previous researches^34,43–46,49–51^. The increased accumulation of lipid in aged cells treated with spermidine confirmed the role of Streptococcal secretions in lipid synthesis and in strengthening the skin barrier function^57,58^. Based on the motif analysis of the upregulated genes, spermidine activates the transcriptional activities of genes related to skin improvement^59,60^.

The increased abundances of *Streptococcus* colonies at the genus level, particularly that of *S. infantis*, were also observed. Spermidine in the St solution positively affected the growths of *S. pneumoniae* and *S. infantis* and this observation suggested that improved skin conditions could positively feedback to themselves through increments of the beneficial skin microbes. In short, our stepwise analysis and findings show that a colonized skin microbiome could improve skin conditions by producing beneficial secretions and suggest that the potential skin microbiome as therapeutic and clinical applications.

In this present study, we have some limitations. In addition to our insight into microbial activity with spermidine, unknown interactions between *Streptococcus* and other bacteria on the human skin could have suppressed the growth of the competitive bacteria for *Streptococcus* candidates, such as the inhibition of *S. aureus* by *Bacillus subtilis* and *Staphylococcus epidermidis*^61,62^. We also have the restricted sex limit to parlay our investigation into the broad applications. Hence, we anticipate that further studies focusing on these details establishing links to skin health and skin microbiome.

## Methods

### Microbial sample collection and preparation

Microbial community samples from the faces of 26 old and 26 young participants were collected using sterilized tape (Elizabeth pack; Cell Lab, Republic of Korea). The tape was then dipped into a liquid Tryptic Soy Broth (TSB) medium (Benton Dickinson, Franklin Lakes, NJ, USA), prepared according to the manufacturer’s instructions. After bacterial growth for 48 h at 37 °C, the medium was centrifuged at 6000 rpm for 10 min. The collected pellet was used to extract microbial DNA with a Quick-DNA™ Fungal/Bacterial Miniprep Kit (Zymo Research, Orange, CA, USA) according to the manufacturer’s instructions. DNA purity and quantity were estimated using a NanoDrop One Spectrophotometer (Thermo Scientific, Waltham, MA, USA).

### 16S rRNA PCR amplification and sequencing

The V3–V4 region of the bacterial 16S rRNA gene was amplified according to the Illumina 16S Metagenomic Sequencing Library Preparation guide (Illumina, San Diego, CA, USA) using the following primers with an added adapter overhang sequence^63^: forward, 5′-TCGTCGGCAGCGTCAGATGTGTATAAGAGACAGCCTACGGGNGGCWGCAG-3′; reverse, 5′-GTCTCGTGGGCTCGGAGATGTGTATAAGAGACAGGACTACHVGGGTATCTAATCC-3′. PCRs were performed in a 25-μL reaction volume containing 2 μL of genomic DNA (10 ng/μL), 0.5 μL of each primer (10 μM), 12.5 μL of 2× KAPA HiFi HotStart Ready Mix (Kapa Biosystems, Wilmington, MA, USA), and 9.5 μL of distilled water. PCR conditions were as follows: initial denaturation at 95 °C for 3 min; 25 cycles consisting of denaturation at 95 °C for 30 s, annealing at 55 °C for 30 s, and extension at 72 °C for 30 s; and a final extension at 72 °C for 5 min. The PCR products were purified with AMPure XP Beads (Beckman Coulter, Brea, CA, USA) according to the manufacturer’s protocol. The attachment of dual-index sequences and Illumina adapters were conducted using 5 μL of the PCR product, 5 μL of Illumina Nextera XT Index Primer 1 (N7xx), 5 μL of Nextera XT Index Primer 2 (S5xx), 25 μL of 2× KAPA HiFi HotStart Ready Mix, and 10 μL of nuclease-free water. Thermocycling was performed as follows: 95 °C for 3 min; 8 cycles of 95 °C for 30 s, 55 °C for 30 s, and 72 °C for 30 s; and a final extension at 72 °C for 5 min. PCR products were purified with AMPure XP beads, and the quality control for the 16S metagenomic libraries was performed using the Agilent Technologies 2100 Bioanalyzer (Agilent, Santa Clara, CA, USA). Libraries were normalized and pooled for sequencing on the MiSeq platform (Illumina) by 2 × 250 bp paired-end sequencing, following standard Illumina sequencing protocols.

### Metagenomic analysis

The quality of the raw sequence reads was analyzed using FastQC^64^. Illumina adapter sequences of the paired-end reads were removed using Cutadapt version 2.2^65^. Then, the trimmed sequences were processed using QIIME2 version 2019.7. Briefly, the reads were assigned to each sample according to a unique index; pairs of reads from the original DNA fragments were merged using an import tool in QIIME2^66^. Quality control and trimming were performed to yield sequences with lengths of 230 and 220 bp for the forward and reverse reads, respectively. To remove low-quality bases at the end of the reads, the DADA2 software package^67^ wrapped in QIIME2 was applied. To remove chimeras from the FASTQ files, a consensus method implemented in DADA2 was used. Beta diversity was compared by principal coordinate analysis using Bray–Curtis distances and weighted UniFrac metrics. Similarity among the groups was evaluated using permutational multivariate analysis of variance (PERMANOVA) with 999 permutations. Taxonomic annotation was performed by mapping the training reference set with primers (forward, 5′-CCTACGGGNGGCWGCAG-3′; reverse, 5′-GACTACHVGGGTATCTAATCC-3′) and extracting the V3–V4 region using GreenGenes version 13_8^68^. Linear discriminant effect size analysis (LEfSe) was performed to identify differential features at the species level between groups based on linear discriminant analysis (LDA) scores using Galaxy implementation^69^. Statistical plots and calculations were generated in R studio with the ggplot2 package^70^.

### Isolation and identification of *Streptococcus* from the face

Sterilized water was used to wash the faces of the participants and was then spread on solid Tryptic Soy Agar (TSA) medium. Single colonies were collected and incubated in liquid TSB medium at 37 °C for 72 h in stationary culture. Each sample was centrifuged at 6000 rpm for 30 min; the pellet was collected, and microbial DNA was extracted with a Quick-DNA™ Fungal/Bacterial Miniprep Kit according to the manufacturer’s instructions. DNA purity and quantity were estimated using a NanoDrop One Spectrophotometer. The bacterial 16S rRNA gene was amplified with the following primers^71^: forward, 5′-AGAGTTTGATCMTGGCTCAG-3′; reverse, 5′-TACGGYTACCTTGTTACGACTT-3′. PCRs were performed in a 25-μL reactor containing 2 μL of genomic DNA (10 ng/μL), 0.5 μL of each primer (10 μM), 12.5 μL of 2× KAPA HiFi HotStart Ready Mix, and 9.5 μL of distilled water. The following PCR conditions were used: initial denaturation at 95 °C for 3 min; 30 cycles of denaturation at 95 °C for 1 min, annealing at 55 °C for 1 min, and extension at 75 °C for 90 s; and a final extension step at 72 °C for 8 min. The PCR products were sequenced on an ABI-3730XL DNA sequencer (Applied Biosystems, Foster City, CA, USA). 16S rRNA fragments were identified using the NCBI Microbial Nucleotide BLAST with Mega-BLAST^72^. The whole-genomic fragments were used to classify the isolated *Streptococcus*.

### Cell culture and St solution treatment

HDFs and HEKs were purchased (PromoCell, Heidelberg, Germany). HDFs and HEKs were cultured in Fibroblast Growth Medium 2 with supplementMix and Keratinocyte Growth Medium 2 with supplementMix, respectively (PromoCell). For St solution treatment, the cells were seeded at 80% confluence into 6-well plates and incubated in an atmosphere of 5% CO_2_ at 37 °C. After 24 h, the cells were washed once with phosphate-buffered saline (PBS) and 10% of conditioned medium was added to the cells together with supplement-free medium, followed by a 24 h incubation.

### Cell viability assay

HDFs and HEKs were seeded in 48-well plates and incubated for 24 h in 1 mL of the complete medium; 10% of the *Streptococcus* culture supernatant was added to the cells; they were incubated for another 72 h. After washing the cells once with PBS, 3-(4, 5-dimethylthiazol-2-yl)-2, 5-diphenyltetrazolium bromide (MTT) solution was added to each well, followed by a 4-h incubation. Then, the medium was discarded, and dimethyl sulfoxide was added to dissolve the formazan crystals. Optical density was measured at 570 nm using a microplate reader and was normalized relative to the untreated control.

### RNA isolation and real-time PCR

Total RNA was isolated from cells using TRIzol reagent according to the manufacturer’s instructions (TaKaRa, Shiga, Japan). cDNA was synthesized from 1 µg of total RNA using Reverse Transcription Premix (Elpis-biotech, Daejeon, Republic of Korea) under the following reaction conditions: 45 °C for 45 min and 95 °C for 5 min. Gene expression was quantified by real-time PCR, and the data were analyzed using StepOne Plus^TM^ software (Applied Biosystems). Real-time PCR amplification reactions were performed using SYBR Green PCR Master Mix with premixed ROX (Applied Biosystems). The following primer pairs (Bioneer, Daejeon, Korea) were used in the reactions inside an ABI 7300 cycler following the manufacturer’s protocol: β-actin (forward, 5′-GGCCATCTCTTGCTCGAAGT-3′; reverse, 5′-GACACCTTCAACACCCCAGC-3′); *COL1A1* (forward, 5′- GAGGGCCAAGACGAAGACATC-3′; reverse, 5′-CAGATCACGTCATCGCACAAC-3′); *COL3A1* (forward, 5′-TGGAGGATGGTTGCACGAAA-3′; reverse, 5′-ACAGCCTTGCGTGTTCGATA-3′); *ELN* (forward, 5′-CACCTTGCCCTTGTAGAATCCA-3′; reverse, 5′-CCATGACAGGTCAACCAGGTT-3′); *FBN1* (forward, 5′- AATGTCAGACGAAGCCAGGG-3′; reverse, 5′-GATTTGGTGACGGGGTTCCT-3′); *DSC2* (forward, 5′- AGTGTGAGTTTGTTCATCACAGGTC-3′; reverse, 5′-CCATGGCCTCACAGCCTTTA-3′); *GBA* (forward 5′-GCTAGGCTCCTGGGATCGAG-3′; reverse, 5′-GTTCAGGGCAAGGTTCCAGTCA-3′); *FLG* (forward, 5′-AGTGCACTCAGGGGGCTCACA-3′; reverse, 5′-CCGGCTTGGCCGTAATGTGT-3′); and *ABCA12* (forward, 5′-ACAGGAATGGCCTTCATCAC-3′; reverse, 5′-AACATGGTGCCCTGAGAAAC-3′). The reaction conditions were as follows: initiation at 50 °C for 2 min and 95 °C for 10 min, followed by cycling at 95 °C for 10 s and 60 °C for 1 min for 40 cycles. β-actin was used as an internal control.

### Nile red staining for neutral lipids

HEKs were seeded in 6-well plates. After 24 h, 10% of the *Streptococcus* culture supernatant was added to the cells, and these were incubated for another 24 h. After washing, a stock solution of Nile red (1 mg/mL) in acetone was prepared and stored at –20 °C away from light. A fresh staining solution was made by adding 1 μL of the stock solution to 1 mL of PBS and then 500-μL aliquots of the mixture were added to each well. After 10 min at room temperature in the dark, the cells were examined using a fluorescence microscope (Axio Observer Z1; Carl Zeiss, Jena, Germany). Neutral lipids were visualized as red fluorescent structures.

### Normal human 3D skin model culture and treatment

A normal human 3D skin model at full-thickness (Epiderm-FT; MatTek Co., Ashland, MA, USA), comprising normal human keratinocytes and normal human fibroblasts, was cultured. The tissue was transferred to 6-well plates and cultured overnight in DMEM (MatTek Co., MA, USA), containing 5 μg/mL gentamicin B (MatTek Co., MA, USA), 0.25 μg/mL amphotericin B (MatTek Co., MA, USA), and other growth factors, in a 5% CO_2_ atmosphere at 37 °C. To induce aging, 3D skin models were treated with poly I:C (1 μg/mL). Afterward, they were treated with St solutions.

### Clinical approval

This study was approved by the Institutional Review Boards (IRBs) of the Global Medical Research Center (IRB: 1-2018060502-A-N-01) and Ellead (IRB: 1-219969-A-N-01).

### Clinical analysis

All steps of clinical analysis were performed in double-blind manners and all individuals were controlled about their cosmetics and cleansers. A total of 22 female healthy participants aged 20–59 years were enrolled in this study after securing their informed consent. On day 1, their basal skin conditions were measured before applying the test solutions. Each volunteer was provided with the control solution (distilled water) and the treatment solution containing 30% St solution (10% *S. pneumoniae*, 10% *S. infantis 1*, and 10% *S. infantis 2*). Each solution was applied to the respective cheeks daily for 4 weeks; measurements were performed on days 1, 14, and 28. Solution application and measurements were carried out at 20–24 °C and 45–55% relative humidity. The following measurements were performed: area of corneocyte (white regions) using ImageJ^73^; brightness of the face using a Mark-Vu instrument (PSIPLUS Co., LTD, Suwon, KOREA) and a continuous light source, data analysis were done using the I-max plus software and expressed as *L*-values^74^; transparency of the cheek using a Translucency meter (TLS850; Dia-Stron, Andover, UK), and the intensity of the scattered light in the skin layer was measured with a fiber optic face plate; TEWL of the cheek using a TEwameter TM300 (Courage + Khazaka electronic GmbH, Köln, Germany) for 25 s; average face moisture using a Corneometer CM825 (Courage + Khazaka electronic GmbH) measured thrice, and the difference in dielectric constants between water and measured area was used; average face elasticity using a Cutometer Dual MPA580 (Courage + Khazaka electronic GmbH) measured thrice; the length of the stretched skin layer following application of a negative pressure (450 mbar), and the unit R2 was defined as Ua/Uf, where Ua is the final retraction and Uf the final deformation^75^; and desquamation of the skin layer was measured by harvesting desquamated skin cells using a D-squame standard sampling disk (D100; Clinical & Derm, Texas, USA) and Visioscan VC98 (Courage + Khazaka electronic GmbH), and the DI was calculated as $D \cdot I = 2A + \mathop {\sum}\nolimits_{n = 1}^5 {{\mathrm{Tn}}(n - 1)} /6$, where A is the percentage of area covered by corneocytes, Tn is percentage of corneocyte related to thickness, and *n* is thickness level; 1–5^76^.

### Genomic analysis

DNA was sequenced using the Illumina HiSeq 4000 sequencer with a read length of 151-bp paired-ends. Libraries were prepared with the TruSeq Nano DNA Kit (Illumina). The sequence reads were trimmed by trimmomatic-0.38 and assembled using SPAdes v3.13.0^77^. Assembled fragments shorter than 100 nucleotides were filtered. Gene prediction was performed using Prokka v1.13^78^ for the assembled and filtered genome. COGs were used to search for common genes between the *Streptococcus* species. A functionally grouped annotation network for the selected genes was constructed using the Cytoscape plug-in ClueGO v2.5.4^79^. ANI was calculated by JSpeciesWS^80^. Functionally related GO terms for biological processes in *Escherichia coli* (version: 18 November 2016) were grouped based on a kappa score >0.4 with a network specificity of 4–10. Statistical significance was calculated using a two-sided hypergeometric test, and the false discovery rate was corrected using the Benjamini–Hochberg method.

### Quantitative analysis of spermidine in St solutions

TSB medium (control) and the St solutions were collected at 1 × 10^8^ CFU/mL and centrifuged at 6000 rpm for 10 min. Then, the supernatants were filtered (0.22 μm membrane filter, Woongki Science Co., Ltd., Seoul, Korea) and a 10-μL aliquot of each, after dilution to 1/10 (TSB medium) and 1/1000 (St solution), was injected to Ultimate 3000 (Thermo Fisher Scientific Inc., Sunnyvale, CA, USA) with the Thermo Hypersil Gold column (50 × 2.1 mm, 1.9 μm, Thermo Fisher Scientific Inc.). The mobile phase [0.1% heptafluorobutyric acid (HFBA) in H_2_O, solvent A; 0.1% HFBA in acetonitrile, solvent B] was eluted at a flow rate of 0.4 mL/min with the elution gradient of 10% (0.01 min) → 10% (0.5 min) → 100% (5 min) → 100% (6.5 min). Next, MS/MS analysis was performed by a Triple TOF 5600^+^ (AB Sciex, Framingham, MA, USA) in the positive-ion mode using electrospray ionization (ESI) source. Spermidine was quantified using multiple reaction monitoring (MRM) with mass transitions from *m*/*z* 146 (Q1) to *m*/*z* 72 (Q3). The operating parameters were set as follows: MS data type, high resolved MS; MS scan type, MRM mode; Ionization source, Electrospray ionization (ESI); Nebulizing gas, 50 psi; Heating gas, 50 psi; Curtain gas, 25 psi; Desolvation temperature, 500 °C; ionspray voltage floating, 4.5 kV; collision gas, 3.5 m Torr.

### Aged-skin cell model and spermidine treatment

Human skin cells (young HDF: neonatal, aged HDF: 60 olds, young HEK: 20 olds, and aged HEK: 40 olds) were purchased from PromoCell (Heidelberg, Germany) and cultured similarly (see *Cell culture and St treatment)*. To stabilize the dose of UVB irradiation, the UVB irradiation machine (CL-1000 ultraviolet crosslinker) was pre-warmed for 30 min. Normal HDF cells were then irradiated with UVB (20 mJ/cm^2^) for 12 s twice a day. After 2 days, the UV-induced aged skin cells were treated with spermidine. Both aged skin cells and UV-induced aged skin cells were treated with 80 μg/mL of spermidine (Sigma, STL, USA). Motif enrichment analysis in the promoters of the upregulated genes was performed by Pscan using the default option^81^.

### Measurement of growth rate

Each *Streptococcus* species was activated in 10 mL of TSB medium at 37 °C while shaking at 200 rpm for 24 h. Then, 10 µL of pre-cultured bacterial cells were separately inoculated in 10 mL of TSB broth and 10 mL of TSB supplemented with spermidine (300 μg/mL) to compare the growth of bacterial cultures. Optical density (OD_600_) was measured by a microplate reader (SPECTRA MAX 190, POWER LAB) every hour for 28 h.

### Statistics and reproducibility

Wilcoxon–Mann–Whitney test was used to calculate the significant differences for non-parametric data. Wilcoxon signed-rank test was used for paired comparison. Pearson correlation was used for correlation analysis. The Student’s two-tailed *t*-test was used in the in vitro cell assay analysis. The power test of the clinical tests was done using 0.7 power. Statistical analyses were performed using R studio or Prism (GraphPad).

### Reporting summary

Further information on research design is available in the Nature Research Reporting Summary linked to this article.

## Supplementary information

Supplementary information

Description of additional supplementary files

Supplementary Data 1

Reporting Summary

## Data Availability

The sequence data that support the findings of this study are available from the European Nucleotide Archive (accession number: ERP116867). Source data underlying plots shown in figures are provided in Supplementary Data 1.
