# Supplementary material for: Spermidine-induced recovery of human dermal structure and barrier function by skin microbiome
Source: Commun Biol. 2021 Feb 19;4:231. doi: 10.1038/s42003-020-01619-4 (PMC7895926; doi:10.1038/s42003-020-01619-4)
Supplement: Supplementary file 1 — Supplementary information [file 42003_2020_1619_MOESM1_ESM.pdf]

Supplementary Information

Spermidine-induced recovery of human dermal structure and barrier function by the skin microbiome

Contents: Supplementary Figures (1-10) and Supplementary Tables (1 and 2)

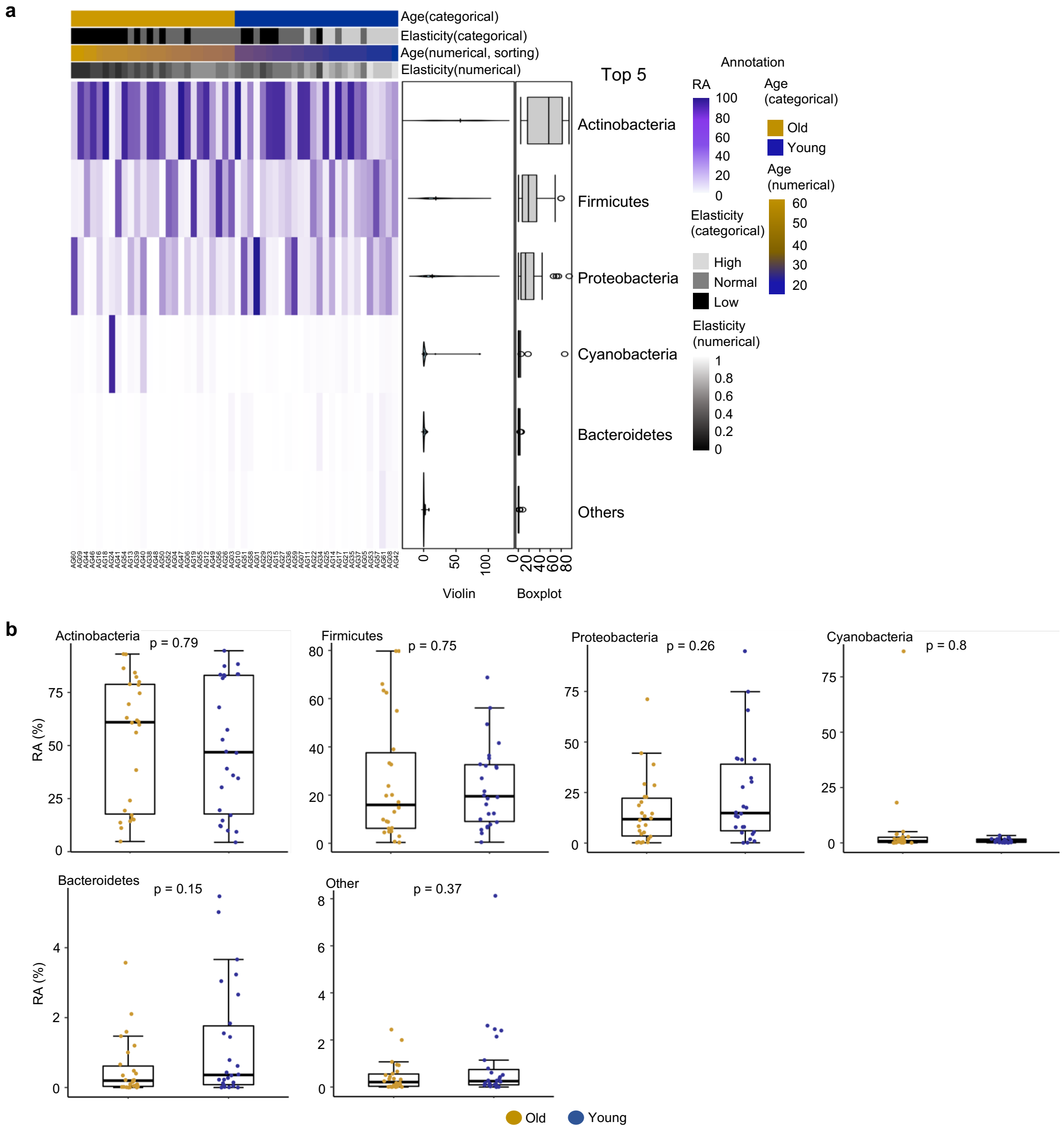

Supplementary Figure. 1 Microbial composition of facial skin samples from old and young participants at the phylum level.

a, Heatmap shows relative abundance (RA) of facial skin microbiome at phylum level with clinical information of skin; the top five abundant phyla are presented; Violin and boxplot shows RA of each skin microbiome; The columns were sorted by ages. **b,** Boxplot describing RA of the top five phyla in old (n = 26) and young (n = 26) individuals. The Mann-Whitney U test was used for statistical significance calculation.

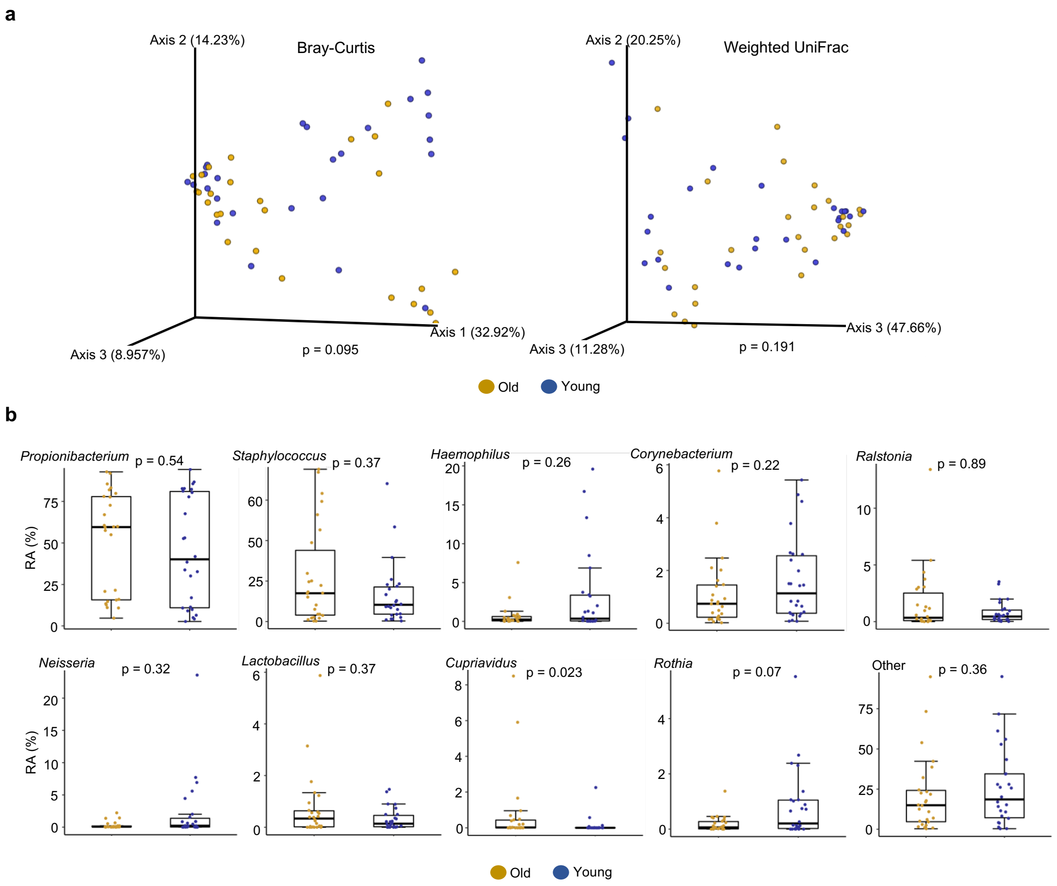

Supplementary Figure. 2 Microbial composition of facial skin samples from old and young participants at the genus level.

a, Beta diversity of the skin microbiome in old (*n* = 26) and young (*n* = 26) candidates. Left, Bray-Curtis distance; right, weighted UniFrac distance. Statistical calculation was done by PERMANOVA with 999 permutations. **b,** Boxplots describing the RA of the top 10 most abundant genera in old and young individuals. The Mann-Whitney U test was used for statistical significance calculation. RA: relative abundance.

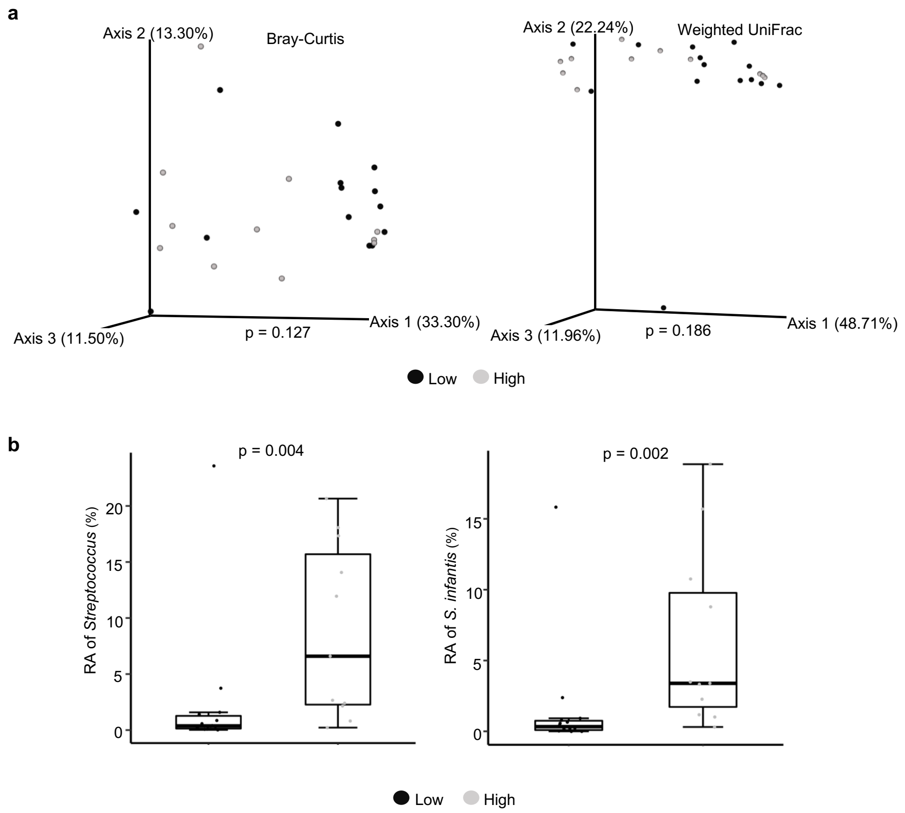

Supplementary Figure. 3 Microbial compositional distance and *Streptococcus* abundances in skin samples with low and high elasticity.

a, Beta diversity of the skin microbiome in individuals with low elasticity (*n* = 14) and high elasticity (*n* = 11). Left, Bray-Curtis distance; Right, weighted UniFrac distance. Statistical calculation was done by PERMANOVA with 999 permutations. **b,** RA of *Streptococcus* and *S. infantis*. The Mann-Whitney U test was used to calculate statistical significance for comparison of abundance. RA: relative abundance.

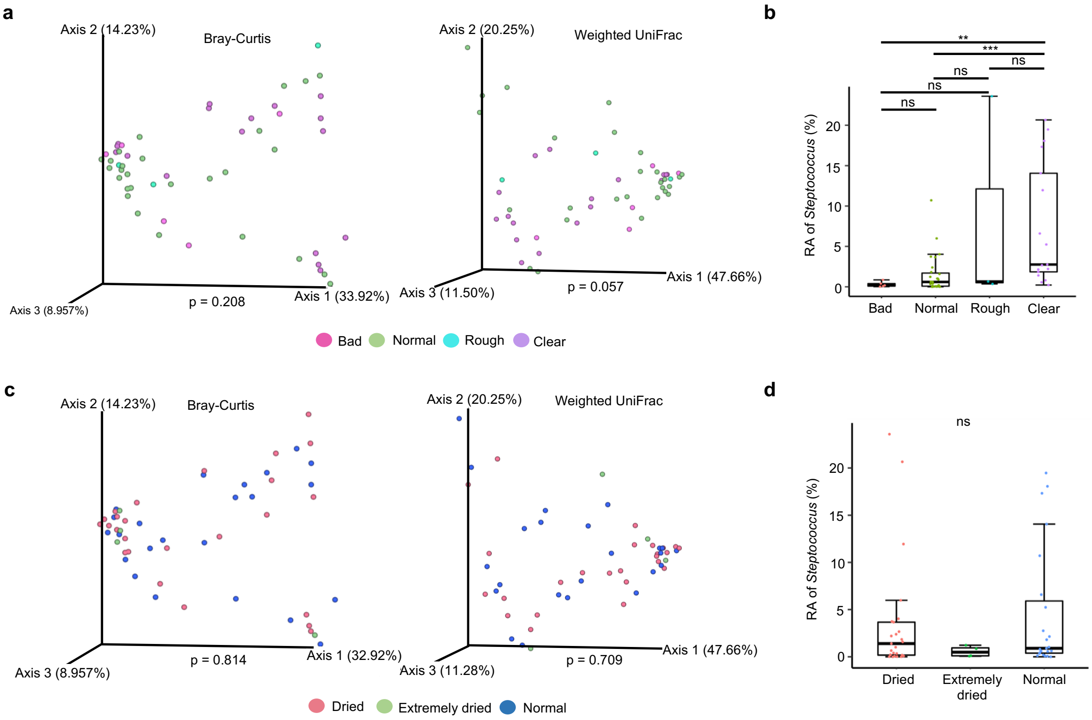

Supplementary Figure. 4 Effect of facial surface conditions on the microbial character of the skin.

a, Beta diversity of the skin microbiome according to different skin appearance conditions: bad (*n* = 5), normal (*n* = 27), rough (*n* = 3), smooth (*n* = 17). Left, Bray-Curtis distance; right, weighted UniFrac distance. **b,** Boxplots describing the distribution of *Streptococcus* in relation to the different facial appearance conditions. **c,** Beta diversity of the skin microbiome under different facial moisture conditions: dry (*n* = 25), extremely dry (*n* = 4), normal (*n* = 23). Left, Bray-Curtis distance; right, weighted UniFrac distance. **d,** Boxplots describing the distribution of *Streptococcus* according to the different facial moisture conditions. RA: relative abundance. Statistical calculation of Beta diversity was done by PERMANOVA with 999 permutations. ns, non-significant. $^{**}p < 0.01$, $^{***}p < 0.001$. The Mann-Whitney U test was used to calculate statistical significance for comparison of *Streptococcus*

abundance. The Kruskal-Wallis test was used to calculated multiple comparison with Benjamini-Hochberg.

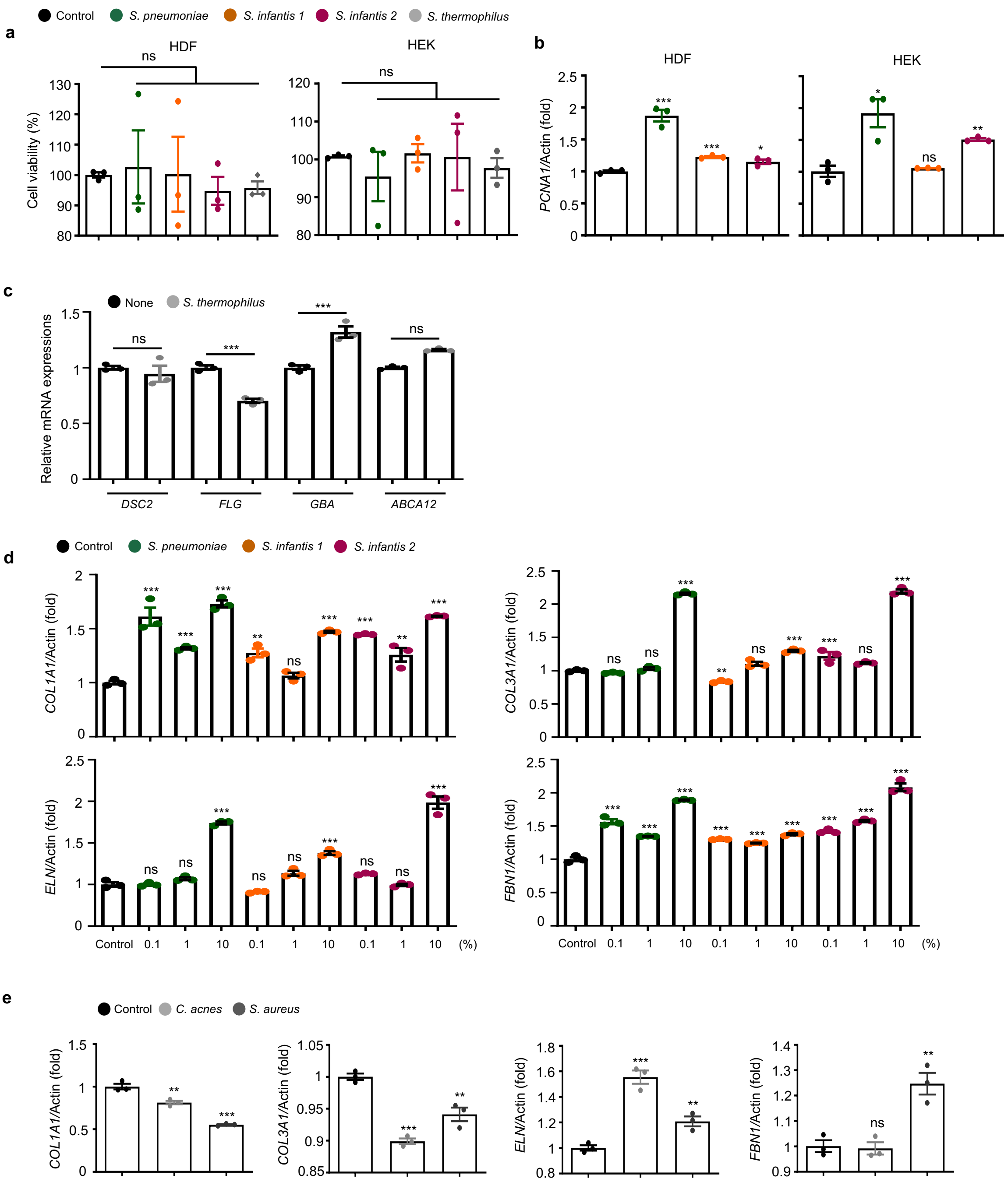

Supplementary Figure. 5 Toxicology and optimal concentration screening of *Streptococcus* culture medium (St solution).

a, Toxicity screening with *Streptococcus* (St) solutions on HDFs (left) and HEKs (right). Each treatment volume of St solution corresponded to 10% of total medium volume. Values represent the mean ± S.E. **b**, Effect of St solutions on mRNA level of *PCNA* (Proliferating cell nuclear antigen). **c,** Effect of *S. thermophilus*-culture supernatant

treatment on mRNA levels of *DSC2*, *FLG*, *GBA*, and *ABCA12*. **d,** Screening test for selecting an optimal concentration of St solution to treat HDFs; mRNA levels of *COL1A1, COL3A1, ELN*, and *FBN1* were used as read-outs. Expression values are relative to none-treated cells and represent mean ± S.E. Student's two-tailed *t*-test was used to calculate statistical significance. *p < 0.05, **p < 0.01, ***p < 0.001. ns, non-significant. Three technical replicates were done. **d,** Effect of treatment with other skin microbiome culture supernatant on mRNA levels of *COL1A1, COL3A1, ELN*, and *FBN1* for HDFs. *COL1A1*, collagen type I alpha 1 chain; *COL3A1*, collagen type III alpha 1 chain; *ELN*, elastin; *FBN1*, fibrillin 1; *DSC2*, desmocollin 2; *FLG*, filaggrin; *GBA*, glucosylceramidase beta; *ABCA12*, ATP binding cassette subfamily A member 12.

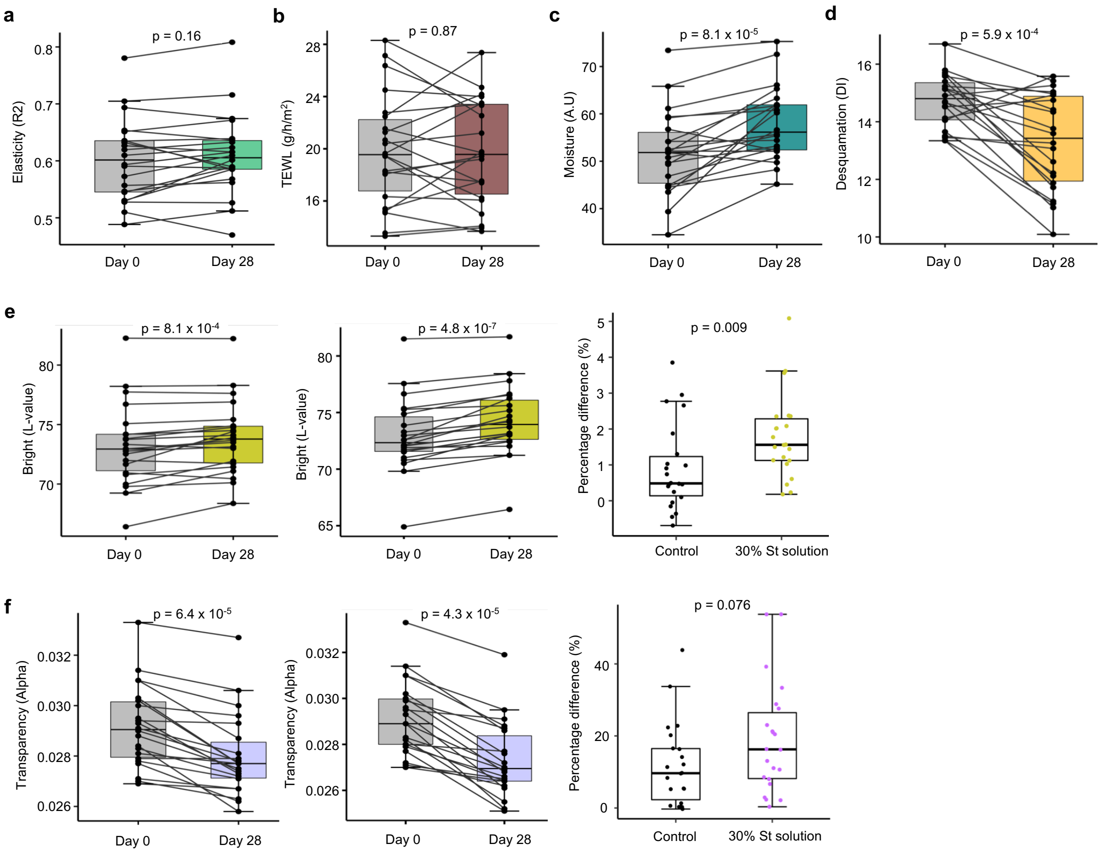

Supplementary Figure. 6 Changes in facial surface conditions following the application of control and St solutions.

a-d, Boxplots illustrating facial skin parameters on day 0 and day 28 following application of control solution. n = 22. **a,** Elasticity. **b,** TEWL. **c,** Moisture. **d,** Desquamation. **e,** Brightness for control solution (left plot), St solution (middle plot), and control emulsion versus St emulsion at final day of treatment (right plot). **f,** Transparency for control solution (left plot), St solution (middle plot), and control emulsion versus St emulsion at final day of treatment (right plot). Statistical calculation for paired comparison was done by the Wilcoxon signed-rank test and inter comparison was done by the Wilcoxon-Mann-Whitney test. A.U, arbitrary unit; DSC, diffuse scattering correction; DI, desquamation index.

a

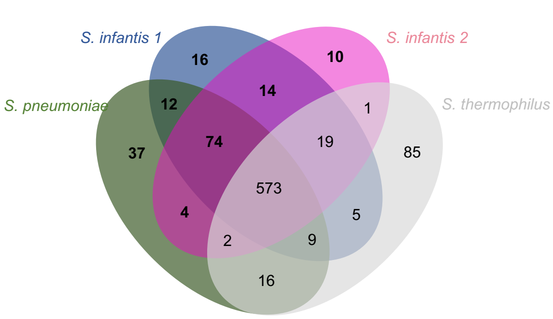

b

	Annotated COGs	Selecting	Genes
S. pneumoniae	727		88
S. infantis 1	722	1. Grouping by genomic distance	73
S. infantis 2	697	2. Matching COGs to genes	72
S. thermophilus	710		82

Supplementary Figure. 7 Whole genomic analysis for selected common genes.

a, COGs of each *Streptococcus* candidate from whole-genome analysis. Common COGs among effective *S. pneumoniae, S. infantis 1* and *S. infantis 2* were indicated by bold. **b,** Procedure for selecting the genes not included in *S. thermophilus* but included in *S. pneumoniae, S. infantis 1*, and *S. infantis 2*. COGs, Cluster of orthologous groups.

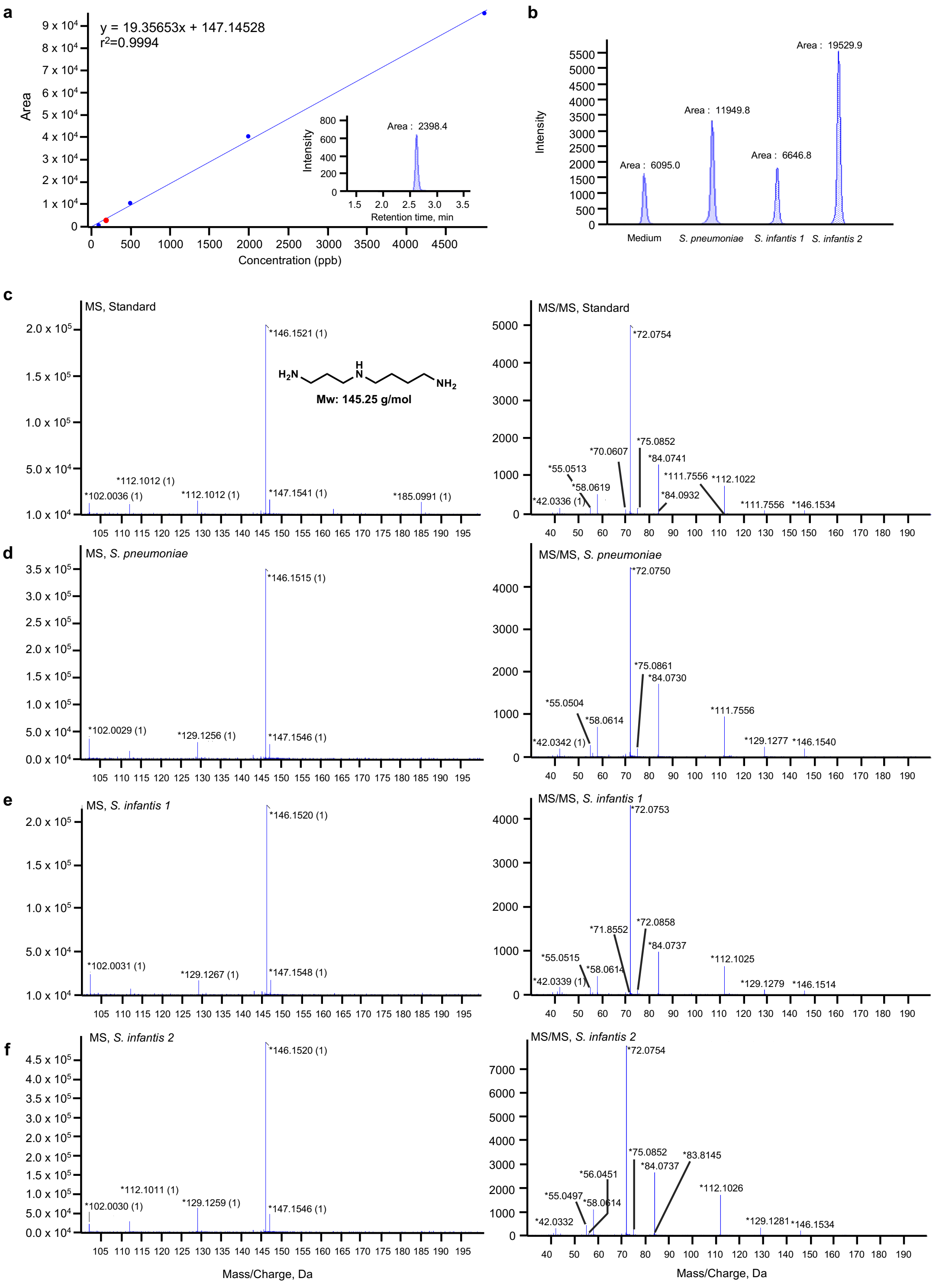

Supplementary Figure. 8 Spermidine concentration in St solutions using UPLC-QTOF-ESI-MS chromatograms.

a, Calibration curve for spermidine which was eluted at 2.68 min among five concentrations. Inserted peak-graph is for red dot. **b,** Spermidine detection at 2.67 ±

0.02 retention time and quantification in medium and St solutions diluted as 1/10 and 1/1000, respectively. The indicated area is used to calculate concentration of the spermidine in each solution. **c-f,** MS (left) and MS/MS (right) peaks of spermidine. **c,** spermidine standard. Molecular structure of spermidine is inserted. **d,** *S. pneumoniae.* **e,** *S. infantis 1.* **f,** *S. infantis 2.*

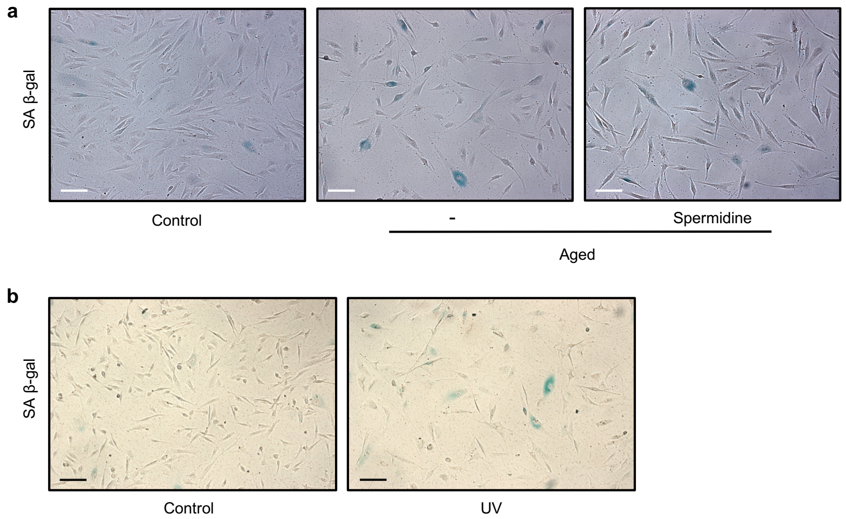

Supplementary Figure. 9 SA β-gal levels in aged cells treated with spermidine.

Microscopic observation of **a,** SA β-gal level of aged skin cells and **b,** UV-induced aged skin cells. The microscopic image was captured with 100X magnification. Inserted scale bar is 100 μm.

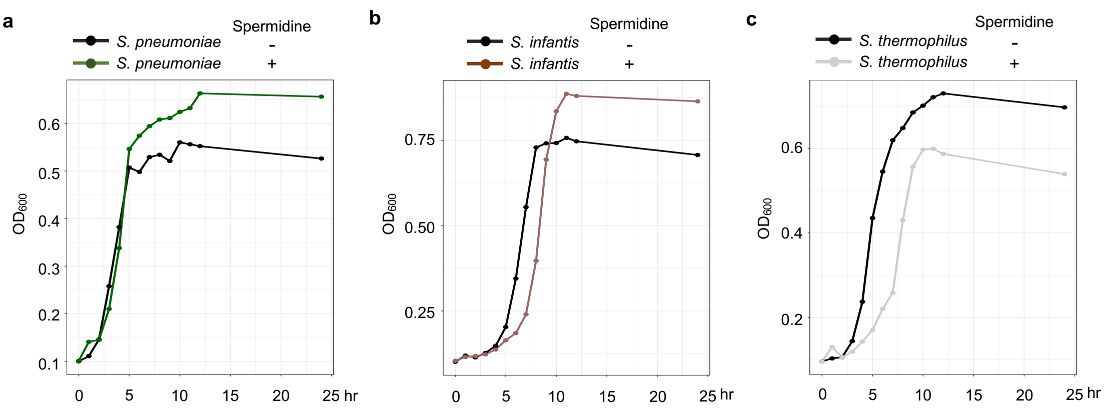

Supplementary Figure. 10 Effect of spermidine on the growth of *Streptococcus*. Growth curves of **a,** *S. pneumoniae*, **b,** *S. infantis*, and **c,** *S. thermophilus* was measured for 24hr with or without the spermidine.

Supplementary Tables

Supplementary Table 1. Summary of participants' characteristics for metagenomic analysis

	Old (n=26)			Young (n=26)			p-value (method)
Age (mean ± SD)	45.5 ± 3.84			24.2 ± 2.87			6.1×10^{-10} (Wilcoxon-Mann-Whitney test)
Moisture (categorical)	Normal (9)	Dried (13)	Extremely dried (4)	Normal (14)	Dried (12)	Extremely dried (0)	0.073 (Fisher's exact test)
Oil (categorical)	Normal (18)	Oily (1)	Oilless (7)	Normal (19)	Oily (1)	Oilless (6)	1 (Fisher's exact test)
Elasticity (numerical, mean ± SD)	0.39 ± 0.14			0.6 ± 0.18			1.3×10^{-3} (Wilcoxon-Mann-Whitney test)
Elasticity (categorical)	High (0)	Normal (14)	Low (12)	High (11)	Normal (13)	Low (2)	3.2×10^{-5} (Fisher's exact test)
Appearance (categorical)	Normal (16)	Smooth (5)	Bad (5)	Normal (11)	Smooth (12)	Rough (3)	0.124 (Fisher's exact test)

Supplementary Table 2. Information on candidates for clinical analysis.

ID	*Age*	*Sex*
S01	35	Female
S02	29	Female
S03	53	Female
S04	44	Female
S05	56	Female
S06	57	Female
S07	46	Female
S08	49	Female
S09	48	Female
S10	50	Female
S11	45	Female
S12	46	Female
S13	41	Female
S14	45	Female
S15	56	Female
S16	43	Female
S17	23	Female
S18	44	Female
S19	51	Female
S20	43	Female
S21	41	Female
S22	51	Female
