# Supplementary material for: Spermidine-induced recovery of human dermal structure and barrier function by skin microbiome
Source: Commun Biol. 2021 Feb 19;4:231. doi: 10.1038/s42003-020-01619-4 (PMC7895926; doi:10.1038/s42003-020-01619-4)
Supplement: Supplementary file 4 — Reporting Summary [file 42003_2020_1619_MOESM4_ESM.pdf]

nature research

Corresponding author(s): Hansoo Park

Last updated by author(s): Nov 10, 2020

Reporting Summary

Nature Research wishes to improve the reproducibility of the work that we publish. This form provides structure for consistency and transparency in reporting. For further information on Nature Research policies, see our Editorial Policies and the Editorial Policy Checklist.

Statistics

For all statistical analyses, confirm that the following items are present in the figure legend, table legend, main text, or Methods section.

n/a	Confirmed	
☐	☒	The exact sample size (*n*) for each experimental group/condition, given as a discrete number and unit of measurement
☐	☒	A statement on whether measurements were taken from distinct samples or whether the same sample was measured repeatedly
☐	☒	The statistical test(s) used AND whether they are one- or two-sided<br>*Only common tests should be described solely by name; describe more complex techniques in the Methods section.*
☐	☒	A description of all covariates tested
☐	☒	A description of any assumptions or corrections, such as tests of normality and adjustment for multiple comparisons
☐	☒	A full description of the statistical parameters including central tendency (e.g. means) or other basic estimates (e.g. regression coefficient) AND variation (e.g. standard deviation) or associated estimates of uncertainty (e.g. confidence intervals)
☐	☒	For null hypothesis testing, the test statistic (e.g. *F*, *t*, *r*) with confidence intervals, effect sizes, degrees of freedom and *P* value noted<br>*Give P values as exact values whenever suitable.*
☒	☐	For Bayesian analysis, information on the choice of priors and Markov chain Monte Carlo settings
☒	☐	For hierarchical and complex designs, identification of the appropriate level for tests and full reporting of outcomes
☐	☒	Estimates of effect sizes (e.g. Cohen's *d*, Pearson's *r*), indicating how they were calculated

Our web collection on statistics for biologists contains articles on many of the points above.

Software and code

Policy information about availability of computer code

Data collection	Illumina sequencing : Illumina Miseq<br>Metabolomic profiling : PeakView 2.2 (Sciex)<br>Measuring skin condition: Mark-Vu instrument, Translucency meter, TEwameter TM300, Glossymeter GL200, Corneometer CM825, Cutometer Dual MPA580, Visioscan VC98 (Detailed information were indicated in Methods)
Data analysis	raw sequence quality control: FastQC v0.11.5<br>OTU taxonomy assignments: GreenGene database (v.13_8)<br>Metagenomic 16S rRNA sequencing data analysis: Qiime2, LEfSe<br>Genome assemmbler: SPAdes v3.13.0<br>Assembly quality assessment: QUAST<br>Bacterial genome annotation: Prokka v1.13<br>Average nucleotide identity (ANI) scores calculation: JSpeciesWS<br>Venn diagrams: jvenn<br>Gene Ontology (GO) analysis: ClueGO v2.5.4<br>Statistical calculation : Prism 8.0 (GraphPad), R studio<br>Difference between two variables : Student's t test, Wilcoxon-Mann-Whitney test, Wilcoxon signed rank test<br>Difference between multiple variables : PERMANOVA with permutations, Kruskal-Wallis test<br>Associations between two continuous variables: Pearson correlation test

For manuscripts utilizing custom algorithms or software that are central to the research but not yet described in published literature, software must be made available to editors and reviewers. We strongly encourage code deposition in a community repository (e.g. GitHub). See the Nature Research guidelines for submitting code & software for further information.

Data

Policy information about availability of data

All manuscripts must include a data availability statement. This statement should provide the following information, where applicable:

- Accession codes, unique identifiers, or web links for publicly available datasets
- A list of figures that have associated raw data
- A description of any restrictions on data availability

The sequence data that support the findings of this study are available from the European Nucleotide Archive (accession number: ERP116867).

Field-specific reporting

Please select the one below that is the best fit for your research. If you are not sure, read the appropriate sections before making your selection.

[x] Life sciences [ ] Behavioural & social sciences [ ] Ecological, evolutionary & environmental sciences

For a reference copy of the document with all sections, see nature.com/documents/nr-reporting-summary-flat.pdf

Life sciences study design

All studies must disclose on these points even when the disclosure is negative.

Sample size	We performed 16S ribosomal RNA gene sequencing on skin microbial samples collected from 52 donors and 22 donors treated with test solution (St solution) for metagenomic profiling (74 donors total).
Data exclusions	no data exclusions
Replication	Experimental findings were reliably reproduced as described in manuscript and figure legends.
Randomization	Clinical measurement with randomization
Blinding	Blinding was performed

Reporting for specific materials, systems and methods

We require information from authors about some types of materials, experimental systems and methods used in many studies. Here, indicate whether each material, system or method listed is relevant to your study. If you are not sure if a list item applies to your research, read the appropriate section before selecting a response.

Materials & experimental systems

n/a	Involved in the study	
[x]	[ ]	Antibodies
[ ]	[x]	Eukaryotic cell lines
[x]	[ ]	Palaeontology and archaeology
[x]	[ ]	Animals and other organisms
[ ]	[x]	Human research participants
[x]	[ ]	Clinical data
[x]	[ ]	Dual use research of concern

Methods

n/a	Involved in the study	
[x]	[ ]	ChIP-seq
[x]	[ ]	Flow cytometry
[x]	[ ]	MRI-based neuroimaging

Eukaryotic cell lines

Policy information about cell lines

Cell line source(s)	Human epithelial keratinocyte (HEK) and Human dermal fibroblast (HDF) were used in vitro assay
Authentication	HEK and HDF were purchased from PromoCell (Heidelberg, Germany)
Mycoplasma contamination	HEK and HDF were negative for mycoplasma contamination
Commonly misidentified lines (See ICLAC register)	Not applicable

Human research participants

Policy information about studies involving human research participants

Population characteristics	74 participants were general women without any skin disease
Recruitment	52 participants were recruited by COSMAX (Seongnam, Korea of republic) and 22 participants were recruited by Ellead (Seoul, Korea of republic)
Ethics oversight	All metagenomic samples and skin conditional data were obtained with informed consent at COSMAX and Ellead. This study was approved by the Institutional Review Boards (IRBs) of the Global Medical Research Center (IRB: 1-2018060502-A-N-01) and Ellead (IRB: 1-219969-A-N-01), respectively.

Note that full information on the approval of the study protocol must also be provided in the manuscript.
